# Generation of micro-droplet arrays by dip-coating of biphilic surfaces; the dependence of entrained droplet volume on withdrawal velocity

**DOI:** 10.1038/s41598-017-12658-z

**Published:** 2017-10-06

**Authors:** Nikolaj Kofoed Mandsberg, Ole Hansen, Rafael Taboryski

**Affiliations:** Department of Micro- and Nanotechnology, Technical University of Denmark, 2800 Kongens Lyngby, Denmark

## Abstract

Droplet array chips were realized using an alignment-free fabrication process in silicon. The chips were textured with a homogeneous nano-scale surface roughness but were partially covered with a self-assembled monolayer of perfluorodecyltrichlorosilane (FDTS), resulting in a super-biphilic surface. When submerged in water and withdrawn again, microliter sized droplets are formed due to pinning of water on the hydrophilic spots. The entrained droplet volumes were investigated under variation of spot size and withdrawal velocity. Two regimes of droplet formation were revealed: at low speeds, the droplet volume achieved finite values even for vanishing speeds, while at higher speeds the volume was governed by fluid inertia. A simple 2D boundary layer model describes the behavior at high speeds well. Entrained droplet volume could be altered, post-fabrication, by more than a factor of 15, which opens up for more applications of the dip-coating technique due to the significant increase in versatility of the micro-droplet array platform.

## Introduction

Creating arrays of chemicals, proteins, and cells is of great interest as it allows for simultaneous monitoring of several reactions^1–4^. Previously, the pipetting technique has been widely used to create such arrays, but recently more sophisticated methods for rapid array creation have been developed^1,2,5–9^. The general tendency has been to break the sequential process of the pipetting technique in favor of a parallel approach in order to save time and effort. This has been achieved by making spatial variance in both surface chemistry and structures^10,11^, so that some superhydrophobic regions are shedding water, while other hydrophilic regions are pinning. The pinning regions get wetted, while the shedding regions stay dry, leaving behind the desired array pattern. The majority of the existing work considers creation of complex droplets and uses techniques mostly suited for proof-of-concept regarding fast formation of droplet arrays^3,6,8,12,13^.

**Figure 1:**
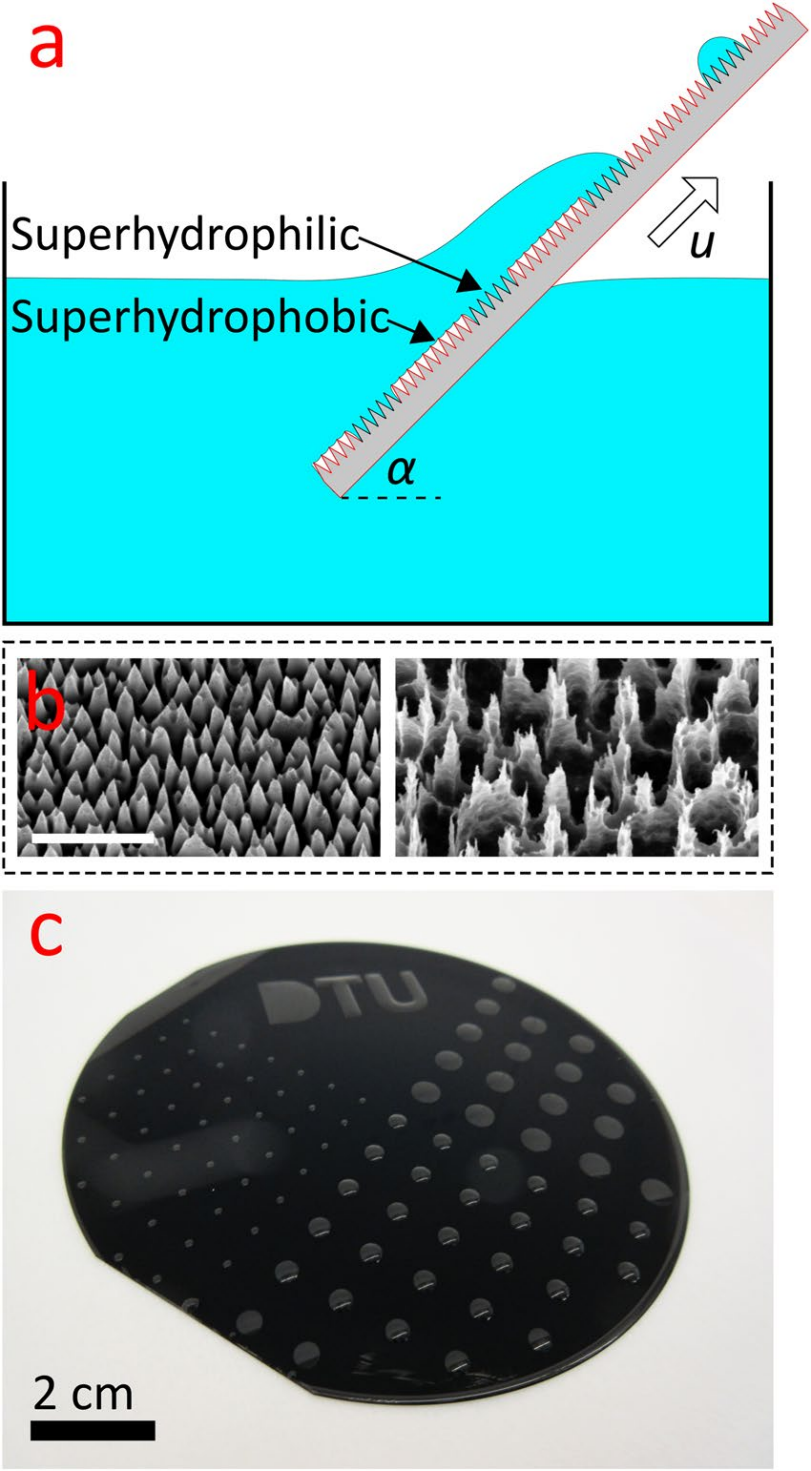
Overview of the experiment, surface nanotexture, and array example. (**a**) Schematic of the experiment showing the array chip being withdrawn from the water reservoir at an angle α with horizontal and at the speed *u*. When submerged, only the ultra-high pinning superhydrophilic regions are fully wetted, while the shedding/superhydrophobic area is protected by an air film. Droplets are present on the pinning spots after the chip has left the reservoir. (**b**) Scanning electron microscopy image of the two types of nanograss surfaces investigated. The scale bar is 1 µm. (**c**) An example of a super-biphilic substrate after submersion and withdrawal. The possibility of creating more complex droplets (“DTU”) is demonstrated.

This paper explores the use of dip-coating to generate micro-droplet arrays. In particular, we studied the properties of the droplets as a function of dip-coating parameters to enable tuning the volume of the created droplets. We fabricated a biphilic surface comprising hydrophilic (pinning) spots on a superhydrophobic area. For these surfaces, we demonstrate how the droplet volume can be altered through appropriate choice of dip-coating parameters. Hence, more specifically we investigate dip-coating of ultra-high pinning circular spots on a superhydrophobic background, which results in well-defined droplets entrained on the pinning spots upon withdrawal from the liquid reservoir. This is done in an experiment, where the velocity by which the array chip is extracted from the water surface at withdrawal is varied. In Fig. 1a the experiment is sketched and the withdrawal velocity, $(u,\alpha )$, is indicated. The surfaces used in this study are nanotextured by employing a reactive ion etching (RIE) method, which can be tuned to make random nano- and submicron structures on silicon surfaces through the combined effect of a corrosive gas (SF_6_) and a passivating (O_2_) gas^14,15^. For simplicity, we denote samples of different SF_6_ and O_2_ flow rates and etching time as ${{\rm{Q}}}_{{{\rm{SF}}}_{6}}-{{\rm{Q}}}_{{{\rm{O}}}_{2}}-{\rm{t}}$. Therefore, 70–50–8 means a sample processed with ${{\rm{Q}}}_{{{\rm{SF}}}_{6}}$ = 70 sccm, and ${{\rm{Q}}}_{{{\rm{O}}}_{2}}$ = 50 sccm for 8 min. Hence, referring to Fig. 1b, the 70–90–8 surface (to the left) and the 70–50–8 surface (to the right) are visualized. As most of the surfaces made this way appear black due to the scattering of the incident light, this method is also known as the “black silicon method”, and has widely been used in optical applications, e.g., as anti-reflective surfaces^15–17^. Figure 1b shows scanning electron microscopy (SEM) images of the two surfaces used here. To obtain a biphilic surface, photolithography was employed to define circular spots of uncoated hydrophilic areas, surrounded by a hydrophobic area coated by a self-assembled monolayer using the precursor perfluorodecyltrichlorosilane (FDTS). This resulted in the high contrast in adhesive properties for the biphilic surface yielding the desired droplet array pattern as exemplified in Fig. 1c.

The use of the dip-coating technique with viscous fluids has been reported by Snoeijer *et al*.^18^ who found the thickness of the film to scale with withdrawal speed to the power 2/3. This agrees with the prediction of Landau and Levich in 1942^19^, who treated the dragging of liquid by a chemically homogeneous plate, having perfect wetting properties and found the limiting film thickness to scale as $h\propto {{\rm{Ca}}}^{2/3}$, where ${\rm{Ca}}=\eta {u}_{0}/\gamma$ is the capillary number, *η* the dynamic viscosity, ${u}_{0}$ the characteristic speed, and *γ* the surface tension. For chemically patterned surfaces (long, narrow hydrophilic lines) Darhuber *et al*. and Davis found a different power law scaling, $h\propto {{\rm{Ca}}}^{1/3}$ ^20–23^. In this study, we find a clear deviation from the classical power law theories, since we observe a finite droplet volume in the zero-speed limit. Moreover, we identify two speed regimes, a low speed regime, where the droplet volume increases with withdrawal speed, and a crossover to a high-speed regime, where volume decreases with speed.

## Results and Discussion

**Figure 2:**
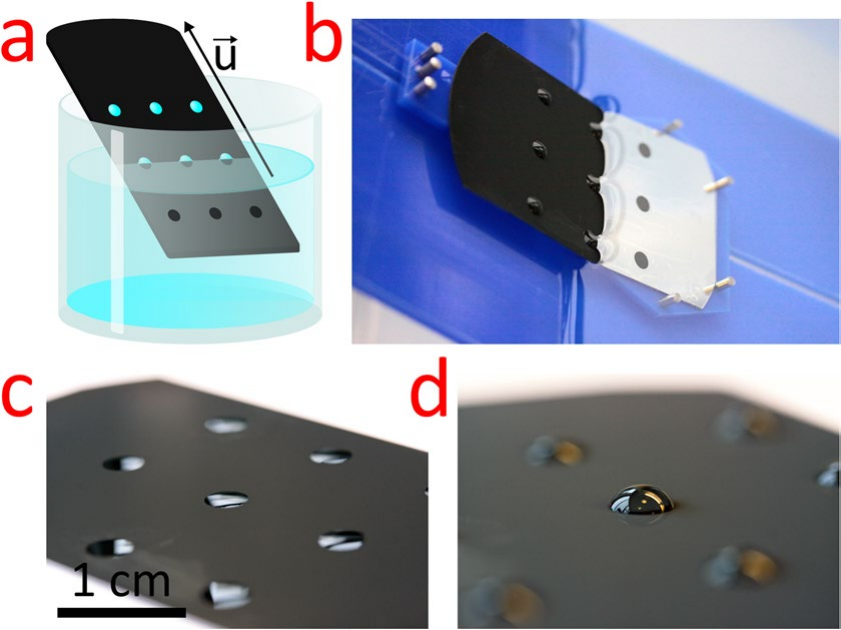
(**a**) Illustration of the dip-coating process used for parallel production of droplets in array. The illustration is showing the chip during withdrawal at a velocity, $\vec{u}$, from the reservoir. At this point droplets have adhered to the ultra-high pinning spots. (**b**) Photograph of the dip-coating process in a situation equivalent to that of (**a**). (**c**) Photograph of the resulting droplet array for a withdrawal speed of 1.4 cm/s and angle of 84°. The single droplet volume is 1.1 µL. (**d**) Photograph of the resulting droplet array for a withdrawal speed of 13 cm/s and angle of 14°. The single droplet volume is 16 µL. For all subfigures the droplet base diameter is ~5 mm.

In this study, we altered the dip-coating withdrawal velocity and measured the entrained micro-droplet mass which was subsequently converted to volume. The dip-coating experiment is illustrated in Fig. 2a. The chip is initially submerged in a reservoir containing the liquid of interest. Withdrawal of the chip at a velocity, $\vec{u}$, causes clearing of the liquid on the superhydrophobic exterior areas, while droplets are left entrained and pinned on the hydrophilic spots. Figure 2b is a photograph from the experimental setup used; the situation is equivalent to that of the illustration. Notice the visual contrast between hydrophilic and hydrophobic areas on the submerged part of the chip. In Fig. 2(c,d) two examples of entrained droplet volumes are visualized. In Fig. 2c the chip, with hydrophilic spots of diameter ~5 mm, was withdrawn at a speed *u* = 1.4 cm/s under an angle *α* = 84° to horizontal. This resulted in droplets of volume 1.1 µL, while in Fig. 2d the chip was withdrawn at 13 cm/s and 14° and gave droplets of volume 16 µL. The ability to alter the droplet volume even after chip fabrication is obvious and here verified by the almost 15 times increase of volume purely by changing velocity. However, as already mentioned, it is not only a matter of speed maximization, since two qualitatively different regimes for the speed-to-volume correlation exist.

**Figure 3:**
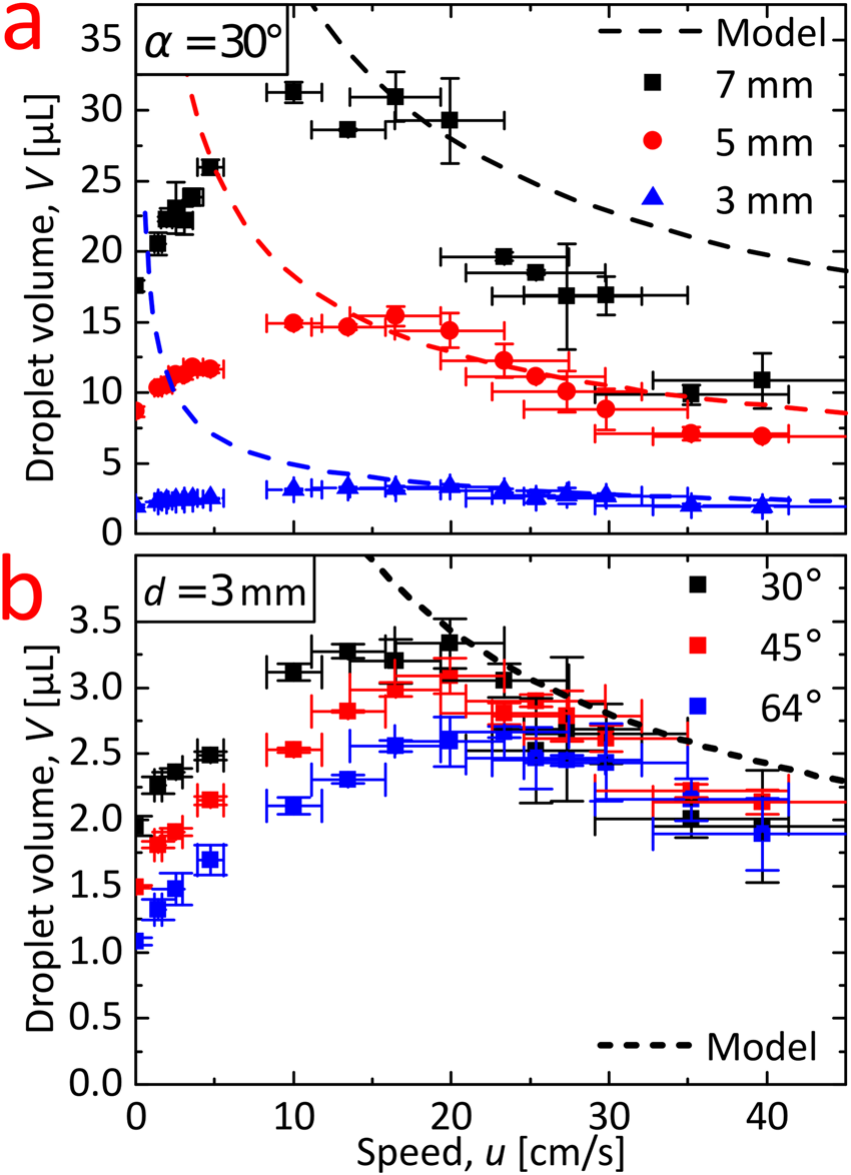
Graphs showing the two regimes for the dependence between droplet volume (uncertainty given as SD, *n *= 2 or 3) and dip-coating withdrawal speed (speed uncertainty given as SD of moving average). The dashed lines are boundary layer predictions by Equation (1) for the high speed regime; where the droplet volume decreases for larger speeds. Experiments were conducted on 70-90-8 nanograss. **(a)** The droplet spot diameter, *d*, varied ((3.14 ± 0.09) mm, (5.3 ± 0.4) mm, (7.3 ± 0.5) mm, uncertainty given as SD, *n* > 850), while keeping the angle of withdrawal, *α*, constant at 30°. **(b)** For a fixed spot size, *d* = 3 mm case, the angle of withdrawal, *α*, is varied.

In Fig. 3a we show the result for speeds from 1.4 to 40 cm/s for three different spot diameters, *d* (3, 5, and 7 mm), while the angle of withdrawal was fixed at 30°. The difference in volume is primarily caused by the spot diameter, but for a particular spot size it is still possible to alter the droplet volume by more than a factor of two by changing the speed at fixed withdrawal inclination angle. Figure 3b is a study with fixed spot size *d* = 3 mm, but with the withdrawal inclination at two additional angles; namely, 45° and 64°. With fixed spot diameter, the variation of the volume measurements is significantly smaller and even vanishes at higher speeds. For low speeds (lower than approximately 16 cm/s), we do, however, observe a dependence of the entrained volume on the angle. For both graphs in Fig. 3, the volume has a maximum value at a finite speed between 10 and 20 cm/s, which defines the transition between two different regimes. The transition was explained by de Ryck and Quéré in 1998 as a transition from a visco- gravitational to a visco-inertial regime with the transition speed obtained at a capillary number ${\rm{Ca}}=1/\sqrt{F}$, where $F=\sqrt{\rho {\gamma }^{3}/g{\eta }^{4}}$, *η* is the dynamic viscosity, *γ* is the surface-tension of water, *g* is the acceleration of gravity, $\rho$ is the density of water, and the capillary number ${\rm{C}}{\rm{a}}=\eta u/\gamma$ ^24^. The de Ryck and Quéré equation results in a transition speed of 16 cm/s in good agreement with our experimental findings. Interestingly, we note that exactly the same transition speed is obtained at unity Froude number, ${\rm{Fr}}={u}_{0}/\sqrt{g{l}_{0}}=1$, where *u*_0_ is a characteristic speed, and *l*_0_ is a characteristic length scale, which we take as the capillary length, ${l}_{0}=\sqrt{\gamma /\rho g}$. Withdrawal at larger angles, *a*, shifts the transition point toward slightly higher speeds (see Fig. 3b and Figure S1 in SI).

Figure 2b shows how the black silicon is ‘black’ above water level as it has anti-reflective properties. When submerged in water the anti-reflective properties are lost in the hydrophobic region; exterior to the spots. The explanation for this change in reflectance is that the superhydrophobicity prevents water from impregnating the surface texture which remain in a Cassie-Baxter wetting state^25^, where an air film is present between substrate and reservoir water (illustrated in Fig. 1a). This gives a significant contrast to the reflection obtained for water in the Wenzel wetting state, which has much lower reflection due to a better refractive index matching between the water and the chip surface^26^. The high adhesion in the Wenzel state, and the low adhesion associated with a true Cassie-Baxter state, suggests that we have no-slip boundary conditions on the droplet-field, and perfect slip in the superhydrophobic areas for the velocity flow field. The viscous boundary layer that develops over the hydrophilic areas is expected to play a crucial role in determining the size of the entrained droplets in the high speed regime^27^. The boundary layer thickness, *δ*, above a flat plate was described by Blasius in the beginning of the 20th century^28^ and can be calculated from:$\,\delta (x)=5\sqrt{vx/u}$, where ν is the kinematic viscosity and *x* is the distance from the front end of the plate; in this droplet formation case we choose *x* as half of the droplet base diameter, *d*. The boundary layer thickness, *δ*, we interpret as the average droplet height, giving an equation that describes the entrained droplet volume, *V*(*u*), at a specific speed, *u*

1 $$V(u)=5\pi \sqrt{\frac{\nu {d}^{5}}{32u}}\,,\,$$

In Fig. 3, the droplet volume obtained from Equation (1) is compared with data. The agreement is good despite the use of the simple 2D model without adjustable parameters. As expected, this model completely fails to describe data for speeds below approximately 16 cm/s since this corresponds to a different droplet formation regime (see Figure S2 in SI for additional data in this regime).

**Figure 4:**
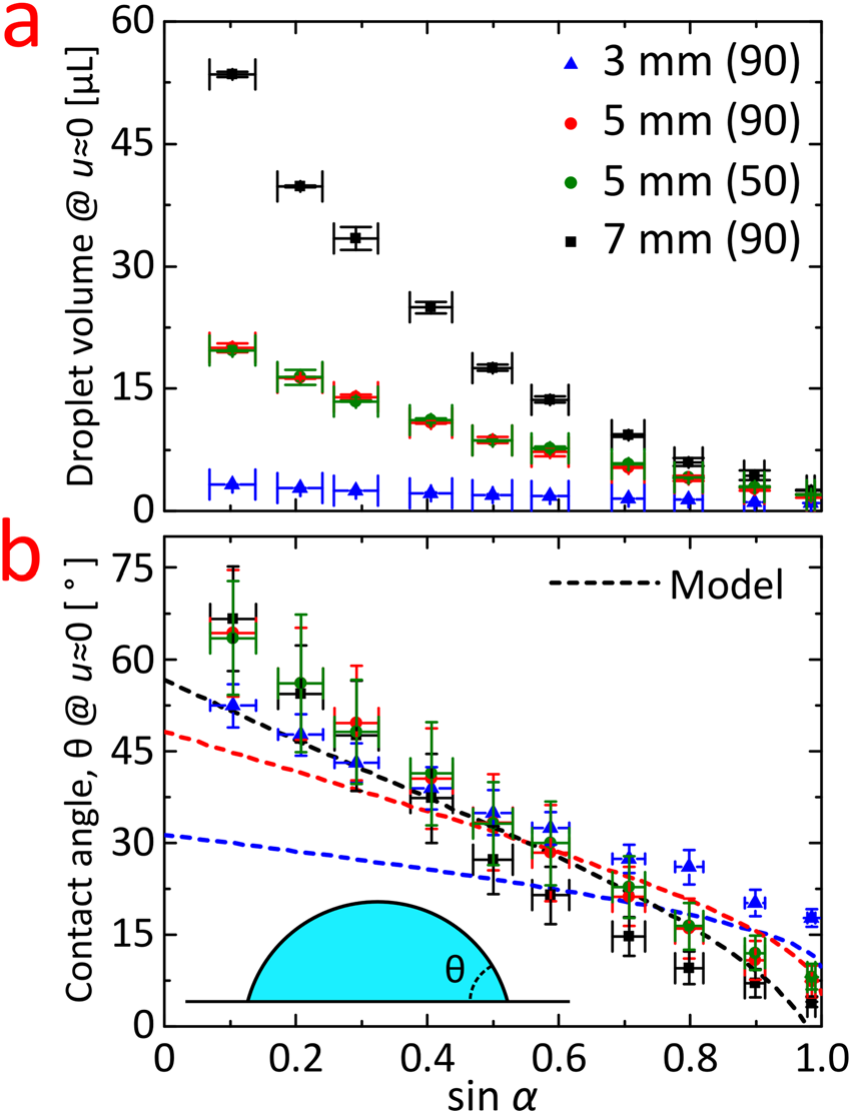
Investigation of the droplet size at very low speed, *u*~1 mm/s. Here the entrained droplet volume is highly dependent on the angle of withdrawal, *α*. The experiment was conducted with spot sizes, *d*, of 3, 5, and 7 mm. For *d* = 5 mm both types of nanograss (see Fig. 1b) were tested. The bracket number (50 or 90) refers to the O_2_ flow in sccm for the nanograss etching process. (**a**) Measured droplet volume, V, as a function of sine to the withdrawal angle. (**b**) Corresponding apparent contact angle, *θ*, vs. sine to the withdrawal angle. The model traces are from the numerically obtained solution to Equation (4).

We now turn to discuss the situation for low withdrawal speed. Figure 4 is the outcome of the withdrawal experiment conducted at very low speed, ∼1 mm/s, for several angles of withdrawal. In addition to experiments with the 70-90-8 surfaces, experiments done with the 70-50-8 surface for the *d* = 5 mm case are also included. In Table 1 we see that the two surfaces 70-90-8 and 70-50-8 are quite different in terms of their wetting behavior, and yet exhibit rather similar performance regarding entrained droplet volume. This indicates that the technique is robust toward choice of the underlying surface roughness. Regarding the contact angle data listed in Table 1, we see that while the 70-90-8 surfaces are truly super-biphilic, having both superhydrophobic and superhydrophilic regions, the 70-50-8 surfaces exhibited a rather high advancing contact angle of (114.1 ± 1.2)° in the pinning regions. Hence, the term “biphilic” here refer to the intrinsic surface chemistry. Figure 4a shows the measured droplet volumes and for the 7 mm spot the volume spans from 2.5 µL to 54 µL by pure angle variation. We notice that all measured droplet volumes are well below (factor of 20-120 for the 7 mm while more for the smaller spots) the sliding instability volume limit caused by contact angle hysteresis at the pinned triple line as obtained from a Furmidge type equation $V=kd{l}_{0}^{2}(\cos \,{\theta }_{r}-\,\cos \,{\theta }_{a})/\,\sin \,\alpha$, where *θ*_*r*_ and *θ*_*a*_ are the receding and advancing contact angles, respectively and *k* is a constant of order 1 depending on the simplifying assumptions made^29–31^.

**Table 1:** Roughness factors and wetting properties of tested types of nanograss. Hydrophilic refers to a silicondioxide surface and hydrophobic to one treated with perflourodecyltrichlorosilane. *θ*_*a*_ is the advancing contact angle and *θ*_*r*_ the receding contact angle.

Structure	Roughness Factor*	Hydrophilic *θ*_*a*_ [°]	Hydrophilic *θ*_*r*_ [°]	Hydrophobic *θ*_*a*_ [°]*	Hydrophobic *θ*_*r*_ [°]*	Hydrophobic Roll-off [°]*
70–50–8	3.2	114.1 ± 1.2	6.4 ± 1.0	167.3 ± 3.0	166.1 ± 3.3	0.9 ± 0.3
70–90–8	4.0	2.7 ± 0.5	≤2.7 ± 0.5**	159.1 ± 2.1	117.5 ± 1.3	27.5 ± 0.9

^*^Data from reference^15^. ^**^Value based on the corresponding advancing contact angle always being larger than the receding.

In absence of flow inertia, the projected gravitational force density, $\rho g\,\sin \,\alpha$, is a key parameter, which is why the entrained volume in Fig. 4a is plotted against $\sin \,\alpha$. The non-linearity suggests that the entrained volume is not governed by a balance of body forces. In Fig. 4b the droplet volumes have been converted to apparent contact angles, *θ*. The conversion is performed using an empirically modified version of Allen’s formula^32^, where we deviate from his requirement of a closed-form conversion formula, but gain a factor of two smaller error. The correction is necessary since Allen’s formula is a *small slope solution* working best for apparent contact angles below 30°. In our modified version, the formula is normalized to the zero-gravity case and acts as a correction factor to the dimensionless shape factor for the spherical cap. The modified equation is given as follows.

2 $$\frac{\tan \,\theta }{\omega }=(\frac{1}{4}\frac{{\beta }^{2}{I}_{1}(\beta )}{\beta {I}_{0}(\beta )-2{I}_{1}(\beta )})(\frac{3\,{\sin }^{4}\,\theta }{{\cos }^{4}\,\theta -3{\cos }^{2}\,\theta +2\,\cos \,\theta }).$$

Here *I*_0_ and *I*_1_ are hyperbolic Bessel functions of the first kind. The modified Bond number $\beta =(d/2)/\sqrt{\gamma /\rho g}$. The dimensionless volume $\omega =8V/\pi {d}^{3}$, where, *V* is the droplet volume. The apparent contact angles of the entrained droplets are plotted as a function of sin *α* in Fig. 4b and exhibit a degree of linearity with only slight deviations for the 7 mm spot. We notice that the actual nanotexturing is without importance for the entrained droplet volumes since both types of tested nanotexture give rise to equal volumes when spot sizes and withdrawal velocities equal; as clearly illustrated by the coincidence of data for the two 5 mm cases in Fig. 4. The presence of finite volumes for vanishing speeds contradicts the usual power law descriptions. The actual relationship between angle and entrained volume could perhaps be explained by the hydrostatic- to Laplace pressure balance. An initial attempt at this considers the pressure balance relating the pressure at the triple line on the superhydrophobic surface to that at the apex of the tilted droplet, evaluated just prior to break-up (see SI Figure S3 for a model sketch). The height *H* of the free liquid surface above the triple line is $H=2{l}_{0}\,\sin \,\frac{{\theta }_{0}-\alpha }{2}$ ^33^, where *θ*_0_ is the apparent contact angle on the superhydrophobic surface, and thus the pressure at the triple line is $\rho gH$. In equilibrium, this pressure must balance with the Laplace pressure at the apex of the droplet (*γC* where *C* is the curvature at the apex) corrected for the elevation of the apex above the triple line (estimated as ${h}^{\ast }\,\cos \,\alpha +a\,\sin \,\alpha$, where *h* ^*^ is the height of the tilted droplet and *a* = *d*/2), i.e.,

3 $$\gamma C+\rho g({h}^{\ast }\,\cos \,\alpha +a\,\sin \,\alpha )=\rho gH.$$

To proceed further a geometrical model for the tilted droplet is needed, which is analytically a very difficult problem (that has not been solved analytically even on a horizontal, flat surface); numerical solutions are of course possible. In the interest of simplicity, we shall proceed with a simple spherical cap model, i.e., we take the curvature $C\cong 2/R=2\,\sin \,\theta /a$ and ${h}^{\ast }\cong h=a(1-\,\cos \,\theta )/\,\sin \,\theta$ where *θ* is the contact angle and *h* the height of the non-tilted spherical cap. Then the simplified pressure balance becomes

4 $$2({l}_{0}/a)\sin \,\alpha +(\frac{1-\,\cos \,\theta }{\sin \,\theta }\,\cos \,\alpha +\,\sin \,\alpha )({l}_{0}/a)=2\,\sin (\frac{{\theta }_{0}-\alpha }{2}),$$

where we have introduced the capillary length *l*_0_. Equation (4) can be solved numerically to obtain the contact angle *θ* as a function of sin (*α*) in the limit of vanishing withdrawal velocity. Considering the simplifying assumptions we have made, only a fair agreement with experiments is expected. Nevertheless, in Fig. 4b calculations (with ${\theta }_{0}=160^\circ$, see SI Figure S4 for ${\theta }_{0}=180^\circ$, which does not affect the trend significantly) are compared to the experimental contact angles as a function of $\sin \,\alpha$. The model mostly underestimates the apparent contact angles but correctly predicts the dependence on $\sin \,\alpha$ where the slope is seen to increase in magnitude with spot size. The discrepancy between model predictions and experiments are probably mostly due to the very crude droplet model and our almost complete ignorance regarding the actual position of the triple line relative to the spot; in fact that position could be a function of both spot size and $\sin \,\alpha$. Even with the limited agreement with experiment, the model adds to our understanding of the system. At slightly higher withdrawal speeds the average droplet volume increases (see Fig. 3 and Figure S2) which is consistent with previous observations^20,22,23,34^. However, due to the very complicated 3-dimensional geometry of the flow limiting region, analytical estimates of the effects of flow speed are very difficult to make. The model also predicts an upper limit for the spot size at a given angle which would allow a finite entrained volume. Whether or not the limit can be reached is, e.g., dependent on the receding contact angle on the hydrophilic spot. More detailed plots in relation to this model can be seen in SI Figure S5.

More complicated droplet geometries beyond the simple circular droplet are certainly possible using this dip-coating technique, and has also been reported in the scientific literature by others^22^. Figure 1c shows an example; in addition to circular superhydrophilic regions of diameter 1, 3, and 5 mm, more complicated regions were created, taking the shapes of letters (DTU). We notice how the “T” and “U” matches the shape of the region, and even allow for negative droplet curvature. Contrary, the “D” has a completely different morphology, where the droplet is ‘closed’ rather than being ‘ring-shaped’. This lens-shape was also obtained by Jokinen *et al*. in 2008^8^ using a pipetting technique, and is of particular interest since it is not a global free energy minimum^35^ and thus shows how local minima can also be exploited to give droplet shapes with more abnormal features.

## Conclusion

In conclusion, we have demonstrated a biphilic micro-droplet array chip and shown how droplet volumes can be controlled through appropriate choices of spot size and withdrawal velocity, i.e., withdrawal speed and angle. In addition, we have demonstrated formation of complicated droplet shapes, that will allow for a wide range of applications^13^, such as synthetization of micro-gels with tailored 3D geometry^36^, and in lab-on-chip diagnostic applications^37^. We have identified two regimes of droplet volume dependence on withdrawal speed. For high speeds, we see a dependence bounded by an expression based on a simple 2D boundary layer model, while for low velocities; we see a clear deviation from the classical power law scaling theories; most notably by the observation of a finite droplet volume at vanishing withdrawal velocities.

The transition between the two regimes can be understood as a transition from the low speed regime dominated by gravity, to the high-speed regime dominated by flow inertia and viscosity. One of the main implications of our findings, e.g., for biochemical micro-array applications is thus the possibility to control the droplet volume in a dip-coating process by simple tuning of the withdrawal velocity. In particular, we found that the droplet volumes could be altered more than tenfold by tuning of the withdrawal velocity in a dip-coating process.

## Methods

### Fabrication

**Figure 5:**
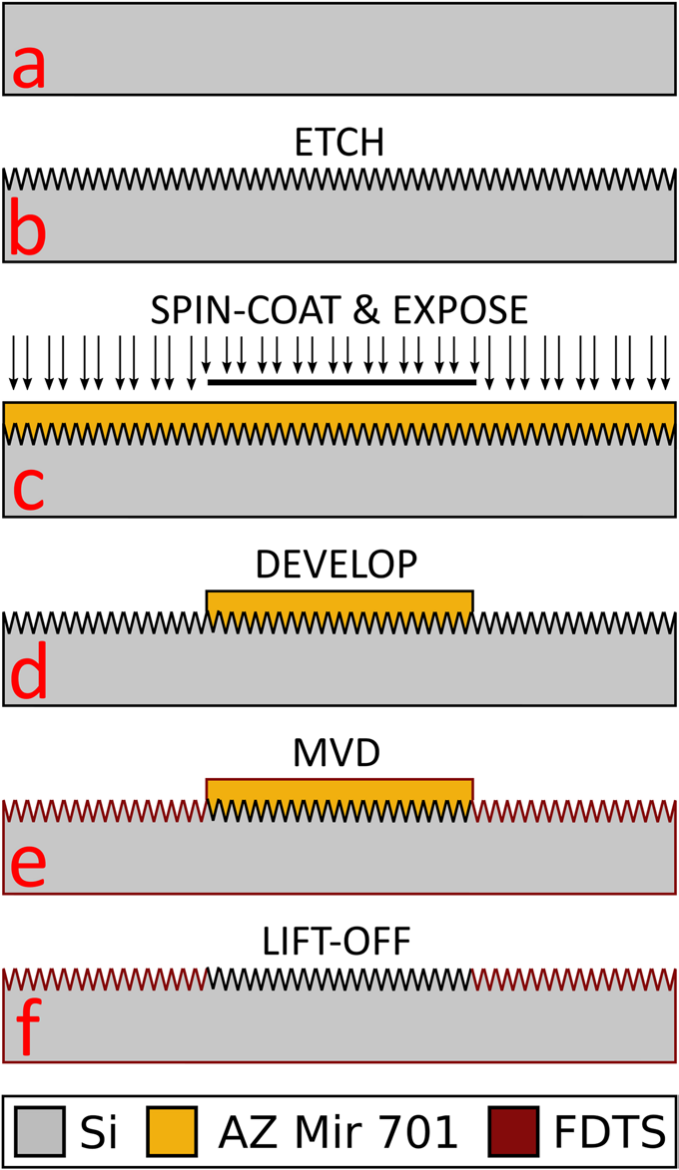
Schematic of the sample fabrication for the biphilic surface structure nanotextured by reactive ion etching (RIE), and chemically patterned with a hydrophobic FDTS self-assembled monolayer. (**a**) Si wafer. (**b**) Reactive ion etch to create surface roughness. (**c**) Spin-coating of positive tone photoresist (AZ Mir 701) and exposure through photomask. (**d**) Development to remove exposed parts of the photoresist. (**e**) Molecular vapor deposition (MVD) of a self-assembled monolayer of perfluorodecyltrichlorosilane (FDTS) to render uncovered regions superhydrophobic. (**f**) Acetone lift-off process to finalize the super-biphilic array chip.

Figure 5(a–f) shows a schematic representation of the fabrication process flow for making the array chips. Biphilic surfaces were achieved by means of a lithographic process whereby a nanotextured silicon wafer with a native oxide surface was coated with a monolayer of a hydrophobic fluorocarbon agent in specific regions. Uncoated regions remained intrinsically hydrophilic. Thus, both regions, hydrophilic and hydrophobic, had the same uniform nanotexture that amplified the adhesive, and non-adhesive, properties, respectively. P-type 100 mm (100) silicon wafers were used for the fabrication (Fig. 5a). Reactive ion-etching (RIE, Pegasus D-RIE, STS, UK) was initially used to create the nanograss nanotexturing, (Fig. 5b). The nanograss recipe from Schneider *et al*.^15^ was used and comprised a mixture of either 90 or 50 sccm O_2_ and 70 sccm SF_6_ gas for 8 minutes; the resulting nanograss structures are denoted 70-90-8 and 70-50-8 surfaces, respectively (see Fig. 1b). The recipes are reported to give different roughness factors (actual surface area to projected surface area)^15^ for the two different O_2_ flows as listed in Table 1. Figure 1b shows Scanning Electron Microscopy images of the two nanotextures. As shown in Fig. 5c, the wafers were then treated with hexamethyldisilazane (HMDS) to enhance photoresist adhesion, and subsequently spin coated (Süss MicroTec Gamma 2 M spin coater) with 1.5 μm positive tone photoresist (AZ Mir 701). The resist was soft baked at 90 °C for 60 seconds. Afterward, the resist was exposed for 30 s at an intensity of 7.0 mW/cm^2^ on a mask aligner (SÜSS MA6) in flood exposure mode through a photo mask placed directly on top of the wafer (see mask design and fabrication details in SI and Figure S6). The resist was then baked at 110 °C for 60 seconds and developed in AZ 726 MIF for 60 seconds (Süss MicroTec Gamma 2 M) (Fig. 5d). Finally, Fig. 5e shows deposition of a self-assembled hydrophobic monolayer using the precursor perfluorodecyltrichlorosilane (FDTS) in molecular vapor deposition (MVD, MVD 100, MST, USA), and Fig. 5f shows lift-off in acetone for 7 minutes followed by a 5-minute rinse in de-ionized water.

### Sample characterization

Figure 1a is a schematic defining the speed, *u*, and angle, *α*, of withdrawal for the super biphilic array chip. Figure S7 in the SI shows a photograph of the setup built to characterize the correlation between the droplet size and the dip-coating velocity. A sledge carrying the 100 mm long array chip was submerged and withdrawn from the 6-8 liter DI-water (Milli-Q) reservoir using two LEGO MINDSTORMS Servo Motors programmed in LEGO MINDSTORMS EV3 Home Edition software. The inclination angle *α* of the slide was adjusted and measured using an electronic inclinometer (Precise Level vs. 2.5, JonyUps, Poland). The associated uncertainty was estimated to 2 degrees. The sledge was submerged into the reservoir, rested for 5 seconds, and was withdrawn again at a certain speed, *u*. The withdrawal speed, *u*, depended on the motor power settings and the chosen gearing. The actual speeds were determined by performing recordings at up to 60 frames per second (fps) using a high-speed camera (PLAYSTATION Eye). From the videos, the time-displacement (*t*, *s*) relationships were obtained using the open source software Tracker 4.9x (see SI, Figure S8 for sample (*t*, *s*) curves). Performing a moving linear regression on 5 data points the local speeds were obtained. Averaging all slopes, obtained from the regressions, gave a best estimate for the speed. The associated uncertainty was calculated as the standard sample deviation based on all the slopes. Due to a high degree of linearity between power settings and determined speed, the speed was only measured for 7 different power settings (see SI, Figure S9 **-**left). Each power setting was run 2–5 times and tested at *α* values of 30° and 60°. Within uncertainty, the speeds were independent of the angle. Speeds at intermediate power settings were obtained from linear interpolation. The relative uncertainties in the speeds were, to a good approximation, independent of the best estimates, allowing prediction of the uncertainty for the intermediate speeds (see SI, Figure S9 **-**right). The determination of the average droplet size was done by creating an array of 4, 9, or 16 droplets. The total mass of the array was measured 30, 40, and 50 seconds after being withdrawn from the reservoir using a precision weighing scale (Sartorius TE214S, 0.1 mg precision). The mass was linearly fitted against time, and the mass at zero time was obtained from extrapolation. For each velocity the dip-coating procedure was performed twice with very high reproducibility. In cases with unusually high uncertainty, a third data point was collected, and confirmed that one of the two initial measurements was an outlier. For each array chip the measurement procedure was initially conducted 10 times to assure reproducible results. Experiments to determine the droplet volume at very low, near-zero, speed were done by steady hand withdrawal and with multiple stops. A very high consistency in the results proves the validity of this latter method.

## Electronic supplementary material

Supplementary Information
