# Supplementary material for: Generation of micro-droplet arrays by dip-coating of biphilic surfaces; the dependence of entrained droplet volume on withdrawal velocity
Source: Sci Rep. 2017 Oct 6;7:12794. doi: 10.1038/s41598-017-12658-z (PMC5630605; doi:10.1038/s41598-017-12658-z)
Supplement: Supplementary file 1 — Supplementary Information [file 41598_2017_12658_MOESM1_ESM.pdf]

Supporting Information for:

Generation of micro-droplet arrays by dip-coating of biphilic surfaces; the dependence of entrained droplet volume on withdrawal velocity

Nikolaj Kofoed Mandsberg, Ole Hansen, and Rafael Taboryski*

Department of Micro- and Nanotechnology, Technical University of Denmark, 2800 Kongens Lyngby, Denmark

* rata@nanotech.dtu.dk

Additional Experimental Data

Withdrawal experiments with speed and angle variation were also conducted for the 5 mm array. The sample was prepared with 70-90-8 nanograss. The data are presented in **Figure S1** and the Blasius model is plotted along with it. A dependence of withdrawal is apparent for low speed but absent for high speed as it is the case for the 3 mm spots (see **Figure 3b**).

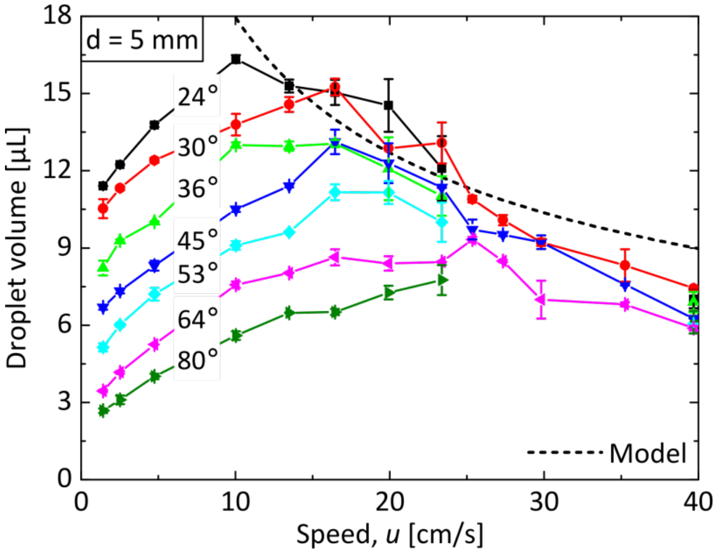

Figure S1*: Graph showing the correlation between droplet volume and withdrawal speed. The droplet base diameter is (5.3±0.4) mm and surface prepared with 70-90-8 nanograss. The withdrawal was done for 7 different angles as indicated in the figure. The dashed line is the Blasius boundary layer model.*

For low speeds the withdrawal experiment was also conducted for various angles with the 70-50-8 nanograss. The results are shown in **Figure S2** and are quantitatively similar to those with 70-90-8 surfaces in **Figure S1**.

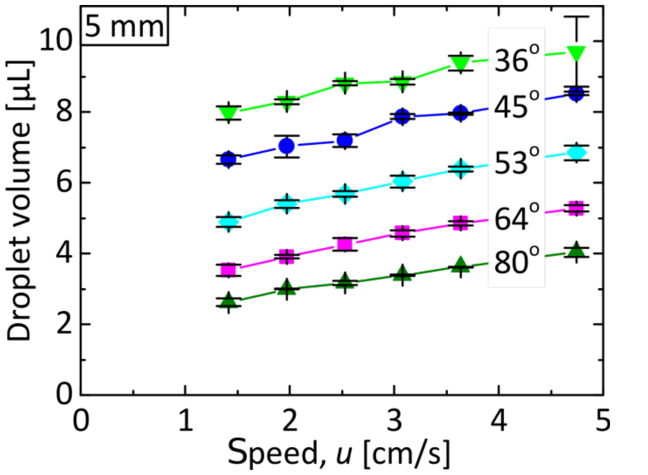

Figure S2*: Investigation of the droplet size at low speeds. Connecting lines are added on the plot to guide the eye. The withdrawal was performed for 5 different angles and 6 different speeds in the interval from 1.1-4.5 cm/s. The spot size is 5 mm and the surface with 70-50-8 nanograss.*

Pressure balance model

In **Figure S3** the sketch used for the derivation of the volume-to-inclination relationship, for zero-speed, is shown.

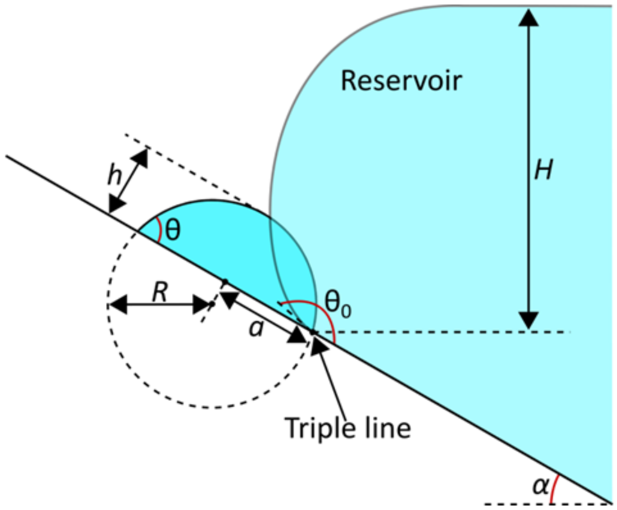

***Figure S3**: Model sketch for the derivation of the pressure balance model. H is the height of the reservoir above the unperturbed triple line of the reservoir. α is the inclination of the withdrawal. θ_0 is the apparent contact angle with the hydrophobic region. a is the distance from the triple line to the symmetry point of the droplet, R the radius of curvature for the entrained droplet, and h the height of the droplet as measured perpendicular to the substrate.*

For superhydrophobic background materials the contact angle is defined to be above 150° and the precision in its measurement decreases as compared to more moderate contact angles. To address possible effects of this uncertainty the developed model has been plotted for both 160 and 180 degrees apparent contact angles, θ_0. The comparison is made in **Figure S4**. Some effect is observed but the overall trend does not change significantly. The slope decreases at smaller spot diameters. The deviation is considered small taking the crudeness of the model into account.

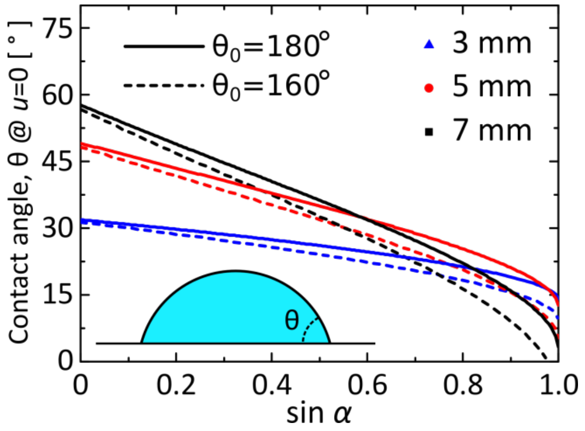

Figure S4*: Pressure balance model for the contact angle of the entrained volume as a function of withdrawal angle. The model is plotted for the hydrophobic region having apparent contact angles of 160 and 180 degrees. The model is shown for the 3 droplet base diameters investigated in this study.*

Figure S5 shows calculated contact angles as a function of normalized droplet base radius a/l_0 obtained from **Equation 4**. The experimental data in **Figure 4b** correspond to radii indicated by the dashed vertical lines in **Figure S5**.

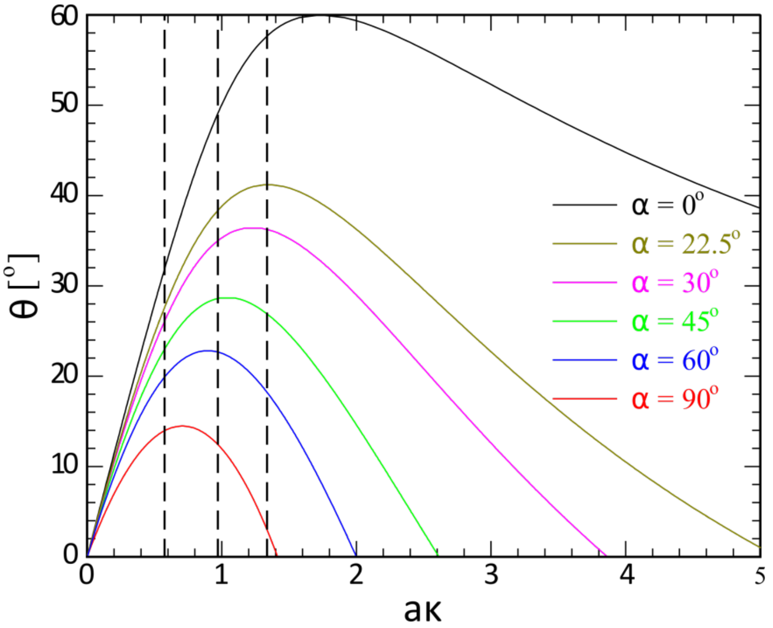

Figure S5*: Pressure balance model with entrained droplet contact angle against spot size for various withdrawal angles. The lower the angle of withdrawal, the larger the entrained volume is. Note,* $\kappa = 1/l_0$.

Array dimensions, mask design, and mask fabrication

The geometrical outline of the array is shown in **Figure S6**. **Figure S6a** includes the full mask design with all the alignment marks for mask printing, UV-exposure, and chip scribing. The array is a square array with a period of four times the hydrophilic spot diameter, d. **Figure S6**(b-c) shows the array outline for the spot sizes of 3 mm and 7 mm.

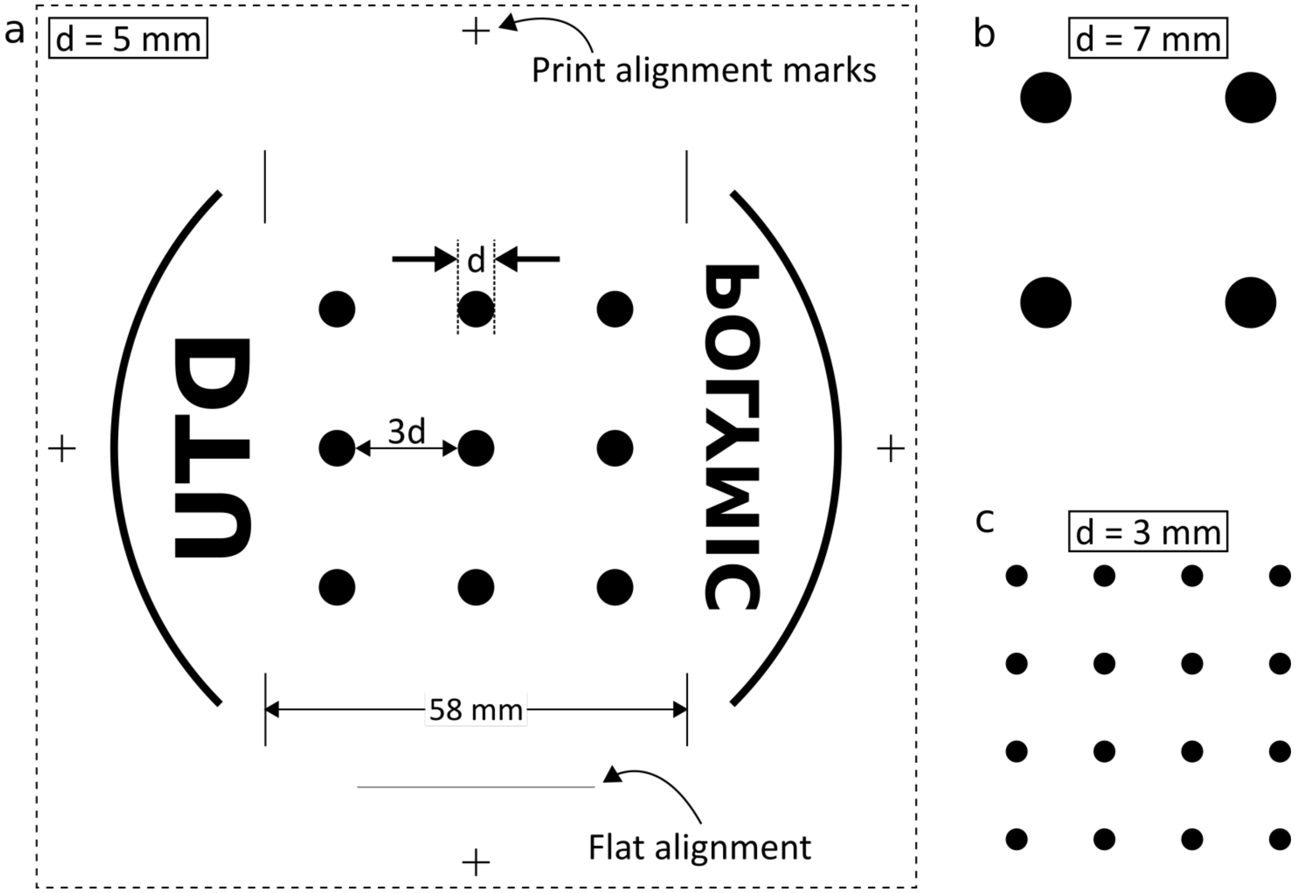

Figure S6*: Droplet array dimensions on the mask used for the photolithographic definition of the hydrophilic spots. a) The complete mask overview for the $d = 5$ mm case. The mask design includes 'print alignment marks' for quick determination of the alignment-quality for the three consecutive prints needed for total blocking of the UV-light. The square array period is 4d and the distance between the scribing marks, for defining the final array chip, is 58 mm. A flat alignment is added to ensure center-alignment of the array to achieve as good nanograss homogeneity as possible.*

<u>Preparation of photo mask</u>

The photo mask was prepared by printing 3 times on top of each other on Premium Transparencies from Xerox with a Xerox 7502V_U printer using the by-pass feeder. An array of non-transparent circles/spots of diameter d on the photomask defines the regions of hydrophilicity. The spots are organized in a square array (see **Figure S6**) aligned with respect to the direction of withdrawal.

Experimental setup

The experimental setup was built and assembled using CO_2 laser cut PMMA (to make the inclined slide and the sledge) and LEGO MINDSTORMS.The pull-chord is fishing line, and the reservoir is 6-8 L of DI water (Milli-Q). The setup is visualized in **Figure S7**.

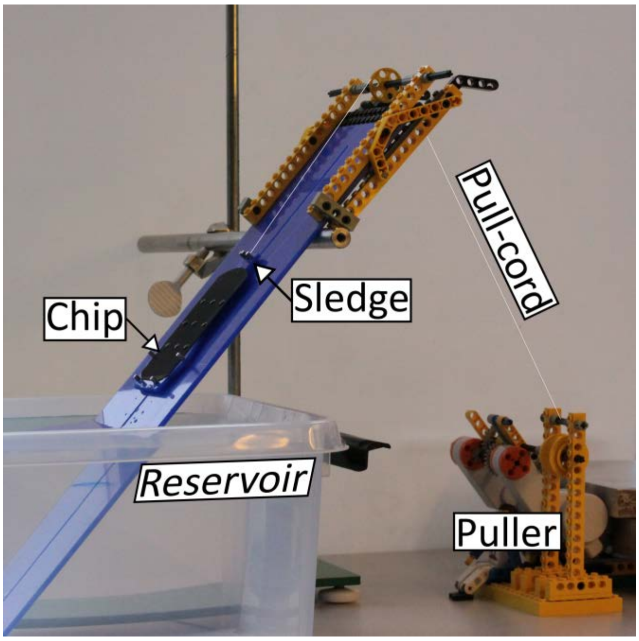

Figure S7*: Photography of the experimental setup. It consists of a blue slide with an elongated opening acting as a trail for the sledge carrying the array chip out of the water reservoir. The sledge is connected to a pulley by a pull-chord, and the pulley is rotated by two programmed servo motor determining the speed of withdrawal. The inclination of the slide is adjustable*

Determination of withdrawal speed

Using a high speed camera (PLAYSTATION Eye) oriented normal to the sledge, the displacement of the sledge was tracked for different motor power settings (see **Figure S8**). L refers to low gearing and H to high gearing. The number, e.g., 20 in L20, refers to the percentage of full motor capacity.

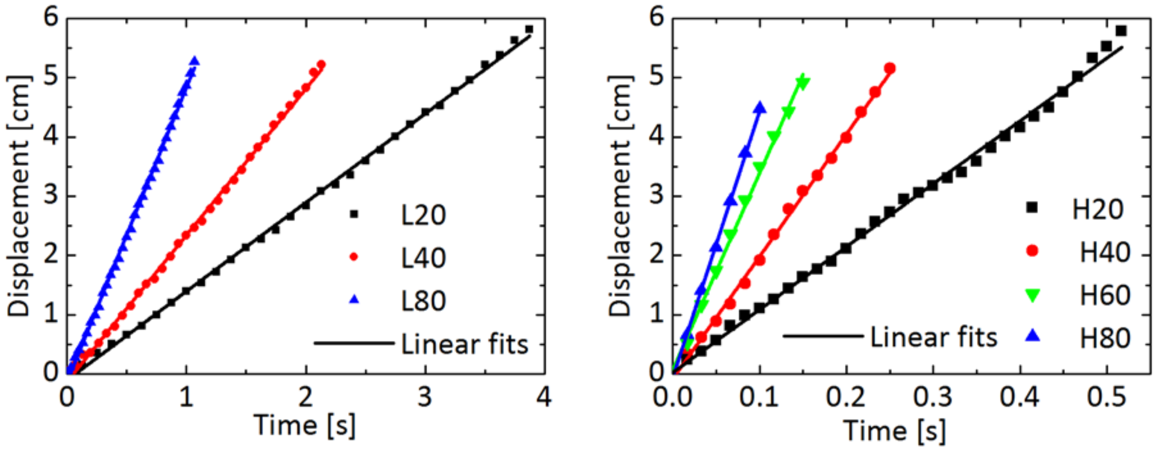

Figure S8*: Sample curves of the sledge displacement as a function of time for different power and gear settings. Data were acquired using a high speed camera and the sledge was tracked using Tracker 4.9x open source software. The local slopes of the graphs are calculated and used to determine the uncertainty in the speed for the setup. (Left) Sledge displacement using low gearing and powers of 20, 40, and 80 % of maximum motor capacity. (Right) Sledge displacement using high gearing and powers of 20, 40, 60, and 80 % of maximum motor capacity.*

The best estimates for the power-speed correlation were determined from the displacement graphs. It was approximated by a linear model as seen in **Figure S9**(left). The associated relative uncertainty was assumed to be constant, 17 %, justified by **Figure S9**(right). For speeds lower than 5 cm/s this is a large overestimate, but to favor the use of a single model for predicting the uncertainty, this has been prioritized.

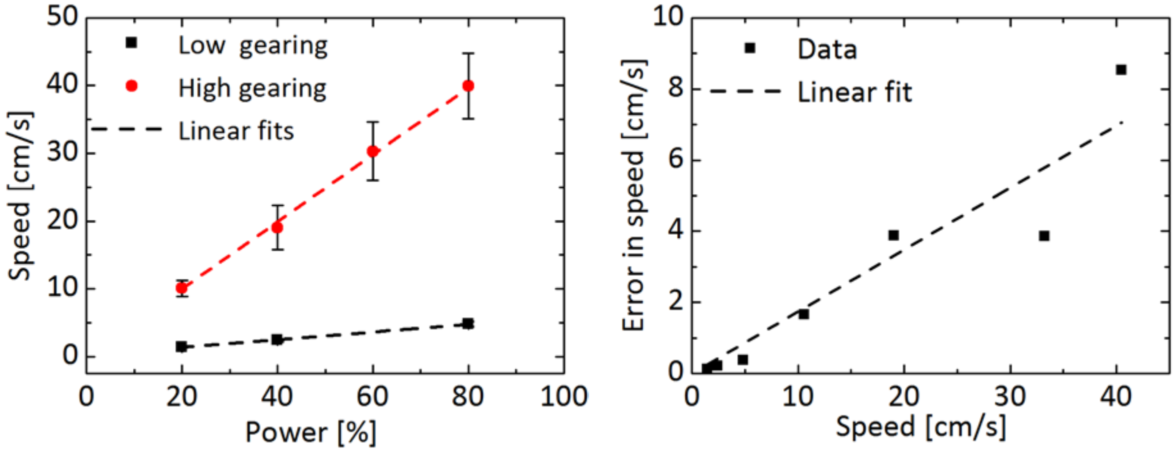

Figure S9*: (Left) Best estimates for sledge speeds for the tested power and gearing settings. The best estimates are based on 2-5 measurements each and the uncertainty is the average internal variation in each measurement combined with the standard sample deviation between the 2-5 measurements. (Right) The associated uncertainty to each best estimate for the speed exhibits a positive correlation that justifies predicting the uncertainty using a proportionality model; hence, stating that the relative uncertainty is independent of the speed.*
